# The relationship between renal function and surgical outcomes of patients with proliferative diabetic retinopathy

**DOI:** 10.3389/fendo.2022.984561

**Published:** 2022-08-25

**Authors:** Jin Liu, Weiwei Zhang, Ping Xie, Songtao Yuan, Lin Jiang, Qinghuai Liu, Zizhong Hu

**Affiliations:** ^1^ Department of Ophthalmology, The First Affiliated Hospital of Nanjing Medical University, Nanjing, China; ^2^ Department of Ophthalmology, The Second Affiliated Hospital of Nanjing Medical University, Nanjing, China; ^3^ Department of Endocrinology, The First Affiliated Hospital of Nanjing Medical University, Nanjing, China

**Keywords:** proliferative diabetic retinopathy, renal dysfunction, vitrectomy, surgical outcomes, Vascular endothelia growth factor-A (VEGF-A)

## Abstract

**Objectives:**

The relationship between renal function and diabetic retinopathy has been controversial. This study is to investigate the influence of renal function on the complex and surgical outcomes of proliferative diabetic retinopathy (PDR).

**Methods:**

This was a *post hoc* analysis of the CONCEPT clinical trial. A total of 45 eyes with PDR underwent vitrectomy were included. Based on the estimated glomerular filtration rate (eGFR), they were divided into abnormal renal function group (*ARF group*) and normal renal function group (*NRG group*). Baseline PDR complex, intraoperative outcomes (Intraoperative bleeding, frequency of endodiathermy, surgical time, iatrogenic hole, and tamponade) and postoperative outcomes (logMAR best-corrected visual acuity, vitreous re-hemorrhage, and macular edema, follow up at postoperative 1 month and 3 months) were estimated. Vitreous, aqueous humor and serum were collected at the vitrectomy day and Vascular endothelia growth factor-A levels were quantified for all included patients using liquid chip method.

**Results:**

There was no significant difference in baseline PDR complex, intraoperative and postoperative outcomes between *ARF group* and *NRG group* (all P > 0.05). At the vitrectomy day, there was also no difference of Vascular endothelia growth factor-A levels in vitreous, aqueous humor and serum between the two groups (all P > 0.05).

**Conclusion:**

Our results showed that the renal function seems not parallel to the severity of PDR, neither to the surgical outcomes. This might be interpreted by the similar Vascular endothelia growth factor-A levels in vitreous, aqueous humor and serum between the two groups.

## Introduction

Globally, more than 415 million people suffer from diabetes mellitus and its complications such as diabetic retinopathy (DR) and diabetic nephropathy (DN). These microvascular complications put a huge burden on health-care systems and decrease patients’ life quality (1, 2). DR is known to be the leading cause of vision loss and even blindness in the working-age population. it has been estimated that the number of people with DR will increase from 126.6 million in 2010 to 191.0 million in 2030, and that 30% of affected individuals require close monitoring or treatment (3). According to American Diabetes Association for type 1 diabetes, the fundus examination should be performed within 5 years after the diagnosis, while for type 2 diabetes, it should be performed at diagnosis. If there is no evidence of diabetic retinopathy for one or more annual eye examination and glycaemia is well controlled, screening can be performed every 1 - 2 years (4). Covid 19 pandemic has made it hard to perform screening programmer, though the role of telemedicine has been further enhanced in particular for retinopathy screening, also using new tools (5, 6). DR begins with asymptomatic retinal abnormalities such as microaneurysms and progresses to proliferative diabetic retinopathy (PDR), the advanced stage which is characterized by neovascularization and preretinal or vitreous hemorrhages (VH) (7). Patients with persistent vitreous hemorrhage (VH), extensive fibrovascular proliferations, or tractional retinal detachment (TRD) are generally indications for pars plana vitrectomy (PPV). Careful evaluation of both the systemic and ocular conditions is of importance for the prognosis of PDR patients with necessary of PPV surgery.

DN, with persistent albuminuria, hypertension, and progressive renal failure (8), is associated with increased morbidity and mortality in diabetic patients (9). The incidence and prevalence of DN have also increased dramatically over the past decade (10). The association between DR and diabetic nephropathy, though has been previously investigated, the relationship between the two diabetic complications has been controversial (11). However, few studies have focused on the influence of renal function on the severity of PDR and the surgical outcomes.

This study aimed to explore the relationship between renal dysfunction and the surgical outcomes of PDR. Besides, we also measured the levels of VEGF-A, the key factor of proangiogenic factor in PDR, in order to interpret our main findings.

## Materials and methods

### Study overview

This was a *post hoc* analysis of the CONCEPT clinical trial, which was registered at https://clinicaltrials.gov/(ID NCT03506750). The study adhered to the tenets of the Declaration of Helsinki, and was approved by Ethic Committee of First Affiliated Hospital of Nanjing Medical University (2017-SR-283). Informed written consent was obtained from all patients prior to enrollment.

In this retrospective study, 45 consecutive eyes of 45 type 2 diabetic patients with PDR between June 2017 and January 2018 were included. Diabetes were diagnosed based on plasma glucose criteria, either the fasting plasma glucose (FPG) value or the 2-h plasma glucose (2-h PG) value during a 75-g oral glucose tolerance test (OGTT), or A1C criteria (12). Criteria for the diagnosis of diabetes included FPG ≥ 126 mg/dL (7.0 mmol/L) or 2-h PG ≥ 200 mg/dL (11.1 mmol/L) during OGTT or A1C ≥ 6.5% (48 mmol/mol) or in a patient with classic symptoms of hyperglycemia or hyperglycemic crisis, a random plasma glucose ≥ 200 mg/dL (11.1 mmol/L) (13). The main inclusion criteria were patients (1) with PDR requiring surgical intervention such as unabsorbed vitreous hemorrhage, tractional retinal detachment; (2) with sufficient intro- and post-operative clinical data for analysis; and (3) with measurement of VEGF-A in serum, aqueous humor, and vitreous. The main exclusion criteria were patients (1) with other neovascular diseases such as retinal vein occlusion, iris neovascularization, or other diseases such as glaucoma, age-related macular degeneration, pathological myopia; (2) with previous intraocular surgery history; (3) receiving preoperative anti-inflammation therapy.

Data collected at baseline included age, gender, duration of diabetes mellitus (DM), PRP history best-corrected visual acuity (BCVA), hemoglobin A1C, and estimated glomerular filtration rate (eGFR). The PDR patients were allocated into two groups, abnormal renal function group and normal renal function group, based on the estimated glomerular filtration rate (eGFR), with the value of 90 mL/min/1.73 m^2^ as cutoff.

### Outcome measures

Outcome measures were the PDR complexity, intraoperative and postoperative complications, and the VEGF levels in aqueous humor, vitreous, and serum at vitrectomy day. PDR complexity were defined as previously reported (14). Intraoperatively, we recorded surgical time, bleeding and endodiathermy frequency, iatrogenic hole, and type of tamponade used. Postoperatively, we recorded BCVA, BCVA at 1 week, 1 months and 3 months, early VH or macular edema (7 days after surgery), and late or macular edema VH (occurred later than 7 days after surgery).

SD-OCT Cirrus (Carl Zeiss, Meditec, Germany) was used to determine the presence or absence of ME, which manifests as diffuse retinal thickening, cystoid macular oedema and serous retinal detachment.

Visual acuities were recorded using a logMAR scale, and visual acuity levels of counting fingers (CF), hand motion (HM), light perception (LP), and no light perception (NLP) were given the following logMAR scores 1.6 logMAR, 2.0 logMAR, 2.5 logMAR, and 3.0 logMAR respectively. Poor vision was defined as a BCVA ≥ 1.6 logMAR (CF) (15).

### Sample collection and measurement strategy for VEGF-A

For all included patients, 0.2 mL undiluted aqueous humor (AQH) was obtained at the start of vitrectomy. Approximately 0.5 mL undiluted vitreous fluid samples were collected with a vitreous cutter at the start of vitrectomy before intraocular infusion. For all patients, serum samples were obtained at the vitrectomy day. Samples were collected into sterile tubes, immediately placed on ice, centrifuged at 1500 rpm for 5 minutes to remove the cells and debris, and then stored at - 80°C until analyzed.

The commercial kits used were Cytometric Bead Array 1 (Bio-Rad) for VEGF-A. The Cytometric Bead Array experiment was contract service offered by Wayen Biotechnologies, Inc (Shanghai, China) with Bio-Plex MAGPIX System (Bio-Rad) in accordance with the manufacturer’s instructions.

### Statistical analysis

Statistical analyses of the data were carried out using SPSS software version 19.0 (SPSS, Inc., Chicago, IL, USA). Values were expressed as means ± standard deviations. Independent t test was used to determine baseline differences in age, duration of PDR and DM, PDR grade, PPV time, and pre-, postoperative BCVA between two groups. Chi-square test was used to determine baseline differences in gender, tamponade, and central macular edema (CME) rate between two groups. Fisher’s exact test was used to determine baseline differences in VH grade, Coagulation frequency and PDR complex score between two groups. The significance of the differences of VEGF-A levels in vitreous, aqueous humor and serum between renal dysfunction group and normal renal function group at the vitrectomy day was calculated by independent t test. *P*<0.05 was considered statistically significant.

## Results

### Baseline data

Forty-five eyes from 45 PDR patients were included in the study. As shown in **Table 1**, there was no difference between the two groups on mean age (p = 0.131), gender (p = 0.408), PDR duration (p = 0.949), DM duration (p = 0.598), Diabetes therapy (Insulin: p = 0.577; Oral hypoglycemic agent: p = 0.620), PDR complex score (p = 0.908) and intravitreal anti-VEGF rate (p = 0.547).

**Table 1:** Baseline characteristics according to renal function category (Renal dysfunction eGFR < 90 mL/min per 1.73 m^2^, normal eGFR > 90 mL/min per 1.73 m^2^).

	Renal dysfunction (n = 20)	Normal renal function (n = 25)	p value
Mean age, years (SD)	53.1(10.36)	47.68(12.71)	0.131
Gender (female)	8(40%)	12(48%)	0.408
PDR duration (month)(SD)	3.16(3.05)	3.1(3.17)	0.949
DM duration (year)(SD	13.28(10.65)	11.76(8.55)	0.598
Diabetes therapy			
Insulin(SD)	8(40%)	13(52%)	0.577
Oral hypoglycemic agent(SD)	13(65%)	13(52%)	0.620
PDR grade(SD)	2.8(2.16)	2.72(2.39)	0.908
Mean pre-operative BCVA (SD) (range)	1.41 logMAR (0.49) (0.22–2)	1.78 logMAR (0.37) (0.82–2.5)	0.006
intravitreal anti-VEGF	15(75%)	21(84%)	0.547

Paired t-test. Chi-square test: Gender, intravitreal anti-VEGF. BCVA, best corrected visual acuity.

### Intraoperative outcomes

All 45 eyes underwent vitrectomy with segmentations and/or delamination. As shown in **Table 2**, there was no difference between the *NRF group* and *ARF* group on intraoperative bleeding mild (p = 1.0), intraoperative bleeding severe (p = 0.767), Frequency of endodiathermy (p = 0.557), PPV time (p = 0.516), iatrogenic hole (p = 0.109) and the tamponade used. For *NRF group*, the preferred tamponade used was silicone oil in 60% (15 eyes) followed by air 40% (10 eyes). For *ARF* group, the preferred tamponade used was silicone oil in 65% (13 eyes) followed by air 35% (7 eyes) (p = 0.767).

**Table 2:** Intraoperative outcomes according to renal function category.

	Renal dysfunction (n = 20)		Normal renal function (n = 25)		p value
	<2’	≧2’	<2’	≧2’	
VH grade	9	11	13	12	0.641
Coagulation frequency	9	11	16	9	0.203
PDR complex score	8	12	12	13	0.591
PPV time(min)(SD)	36.95 (21.82)		42.68(33.9)		0.516
iatrogenic hole	0		3		0.109
Tamponade (%)					
Air		6(30%)	10(40%)		0.486
Silicone		13(65%)	15(60%)		0.731
Gas		1(5%)			0.258

Chi-square test. Fisher’s exact test. VH, vitreous haemorrhage.

### Postoperative visual outcomes

As shown in **Table 3**, there was no difference between the *NRF group* and *ARF* group on mean BCVA at 1 postoperative week (p = 0.615), 1 month (p = 0.404) and 3 months (p = 0.115).

**Table 3:** Postoperative BCVA according to renal function category.

	Renal dysfunction (n = 20)	Normal renal function (n = 25)	p value
Mean BCVA at 1 week (SD) (range)	1.05 logMAR (0.59) (0.4–2.5)	1.13 logMAR (0.47) (0.4–2)	0.615
Mean BCVA at 1 month (SD) (range)	0.93 logMAR (0.48) (0.4–2)	0.81 logMAR (0.47) (0.3–2)	0.404
Mean BCVA at 3 month (SD) (range)	0.99 logMAR (0.54) (0.3–2)	0.74 logMAR (0.5) (0.1–2)	0.115
Mean difference in BCVA (SD)	0.42 logMAR (0.58)	1.07 logMAR (0.61)	0.001

Paired t-test. VH, vitreous haemorrhage.

Visual improvement by at least 0.3 logMAR units occurred in 33 eyes (73.3%), 4 (8.9%) improved by less than 0.3 logMAR units, and 4 (8.9%) worsened by at least 0.3 logMAR units. 20% of eyes (6.7% were in the *ARF* group and 13.3% were in the *NRF* group) were recorded with BCVA of 6/12 (0.3 logMAR) or better at 3 months.

### Postoperative complications

VH occurred in 15.6% (n = 7) of all included patients. 11.1% (n=5) were with early and 4.4% were with late hemorrhage (n=2). The presence of early or late VH postoperatively was not different between the *ARF group* and *NRG group* (**Table 4**).

**Table 4:** Postoperative outcomes according to renal function category.

	Renal dysfunction (n = 20)	Normal renal function (n = 25)	p value
Early VH(%)	2(10%)	3(12%)	0.832
Late VH(%)	1(5%)	1(4%)	0.872
Early CME(%)	6(30%)	13(52%)	0.138
Late CME(%)	10(50%)	17(68%)	0.221

Chi-square test. VH, vitreous haemorrhage; CME, cystoid macular edema.

CME occurred overall in 42.2% (n = 19) at postoperative 1 month and 60% (n = 27) at postoperative 3 month. The presence of neither early nor late CME was associated with the renal function category (**Table 4**).

### VEGF-A levels in vitreous, aqueous humor and serum at the vitrectomy day

Intravitreal anti-VEGF was used in 36 eyes (80%). For the 36 eyes, there was no statistically significant difference of VEGF-A level in vitreous, aqueous humor and serum between renal dysfunction group and normal renal function group at the vitrectomy day (**Table 5**).

**Table 5:** VEGF-A levels in vitreous, aqueous humor and serum between renal dysfunction group and normal renal function group at the vitrectomy day.

	Renal dysfunction (n = 15)	Normal renal function (n = 21)	p value
vitreous(SD)	54.33 (20.65)	59.54 (30.15)	0.567
aqueous humor(SD)	17.58(3.04)	21.72 (17.19)	0.365
serum(SD)	78.44(168.78)	61.88(99.82)	0.714

Paired t-test.

## Discussion

The present study shows that there was no significant difference in baseline PDR complexity, intraoperative and postoperative outcomes between *abnormal renal function* and *normal renal function* groups (all P > 0.05). The presence of renal dysfunction seems not affect surgical complexity and the prognosis of the PDR patients undergoing vitrectomy for PDR, which can be explained by the similar expression level of VEGF-A in patients’ vitreous, aqueous humor, and serum.

Diabetic retinopathy (DR) is a major global health burden due to its high prevalence and associated high risk of vision impairment (16). Chronic kidney disease (CKD), also known as one of the most common complication of diabetes, shows certain similar pathogenesis with DR. Chronic exposure to hyperglycaemia affects the microvasculature, eventually leading to diabetic nephropathy and retinopathy (17). Both the two types of diabetic microangiopathy involve thickening of basement membrane and muscular layers and increased leakage. Furthermore, a recent study has suggested common inherited susceptibilities to retinopathy and CKD in diabetic patients (18).

The association between CKD and DR has been extensively investigated in previous studies (19). A number of studies provide evidence that DR may be independently associated with the development of micro albuminuria and, hence, be a powerful predictor for the progression of renal damage in DM patients (20). Albumin excretion rate (AER) was significantly higher among subjects with either PDR or severe NPDR as compared with mild NPDR (21). But most were about DR at early stage or without staged. Other investigations show that the progression of DR and DN for patients with T2D was discordant. The Renal Insufficiency and Cardiovascular Events study found that the progression of DN did not affect 41.4% of T2D patients with advanced DR (22). However, few studies have investigated the influence of renal function on the severity of PDR and the surgical outcomes.

Postoperative VH and CME are common complications after vitrectomy for PDR (23, 24). Gupta et al. found, in a similar cohort of patients, a prevalence of VH of 31.9% in patients with TRD (25). In our series, 15.6% of patients developed postoperative VH. CME occurred overall in 42.2% (n = 19) at postoperative 1 month and 60% (n = 27) at postoperative 3 month. Early or late VH and CME were not significantly different among the 2 renal categories. Our visual results also show that there was no significant difference in mean BCVA postoperative 3 months between renal dysfunction group and normal renal function group. All above can be explained by the similar expression level of VEGF-A in patients’ vitreous, aqueous humor, and serum.

Chronic kidney disease has been implicated in the production of vascular endothelial growth factors (VEGF) that are normally detected in podocytes in glomeruli and tubular epithelial cells (26, 27). A marked expression of VEGF secondary to glomerular injury has been observed in experimental diabetic rats with early nephropathy (28). Local production of VEGF may be further released into the systemic circulation. Our results showed that the renal function seems not parallel to the severity of PDR, neither to the surgical outcomes. This might be interpreted by the similar VEGF-A levels in vitreous, aqueous humor and serum between the two groups at the vitrectomy day. Previous study has shown that the humoral VEGF-A level was significantly lower at vitrectomy day than injection day, indicating that anti-VEGF injections preoperatively are a useful surgical tool to reduce postoperative vitreous cavity hemorrhage (29).

Limitations of the study include its retrospective nature and the inherent difficulties of retrospective data collection including the identification of confounders, which cannot always be adjusted for in the analysis. Secondly, the sample size of this study is relatively small. Prospective studies on a larger number of eyes with a longer follow-up period are required. Thirdly, the correlation between the severity of renal and retinal damage is much closer in type 1 diabetes than in type 2. All patients in our study were type 2 diabetic patients with PDR. The PDR patients were allocated into two groups, abnormal renal function group and normal renal function group, based on the estimated glomerular filtration rate (eGFR), with the value of 90 mL/min/1.73 m^2^ as cutoff, which might be improper. Fourthly, other evaluation indices of renal function such as urine protein were absent. Lastly, as for diabetes therapy, there was no significant difference in either insulin or oral hypoglycemic agents between the two groups. The oral hypoglycemic agents the patients used in this study included biguanide, sulfonylureas, acarbose and glinides. Some antidiabetic drugs such as sodium-dependent glucose transporters 2 (SGLT-2) inhibitor may have a nephroprotective role that could influence the correlation between the severity of the two types of diabetic microangiopathy. The patients in the current study did not use this drug mainly because SGLT-2 inhibitor has been on the market in China since the end of 2017, while our research was carried out between June 2017 and January 2018. The influence of nephroprotective antidiabetic drugs on the relationship between renal function and diabetic retinopathy should be further investigated.

In conclusion, there was no difference of pre- and post-operative outcomes between PDR patients with and without renal dysfunction, which can be induced by no significant difference in VEGF-A level in vitreous, aqueous humor and serum between the two groups. Renal function alone should not be considered a prognostic marker for PPV surgery for patients with PDR.

## Data availability statement

The raw data supporting the conclusions of this article will be made available by the authors, without undue reservation.

## Ethics statement

The studies involving human participants were reviewed and approved by the Ethic Committee of First Affiliated Hospital of Nanjing Medical University. The patients/participants provided their written informed consent to participate in this study. Written informed consent was obtained from the individual(s) for the publication of any potentially identifiable images or data included in this article.

## Author contributions

We declare that all authors have made substantial contributions. JL, WZ, PX, SY, LJ, QL, and ZH conceived the study, developed the protocol, and supervised the study. JL, WZ, and PX collected the data. SY and LJ performed the preliminary data analysis. JL, WZ, and SY performed the final data analysis. All authors contributed to the conduct of the study and interpretation of results. JL, WZ, QL, and ZH drafted the manuscript and all authors contributed to critical revisions of the manuscript. All authors read and approved the final manuscript.

## Funding

This study was supported by Natural Science Foundation of Jiangsu Province (No. BK20191059) and National Key Research and Development Program of China (No. 2017YFA0104100 to QL).

## Acknowledgments

We thank all the patients who participated in our investigation and those who have helped us in the research but not mentioned as co-authors.

## Conflict of interest

The authors declare that the research was conducted in the absence of any commercial or financial relationships that could be construed as a potential conflict of interest.

## Publisher’s note

All claims expressed in this article are solely those of the authors and do not necessarily represent those of their affiliated organizations, or those of the publisher, the editors and the reviewers. Any product that may be evaluated in this article, or claim that may be made by its manufacturer, is not guaranteed or endorsed by the publisher.
